# Vitamin C controls neuronal necroptosis under oxidative stress

**DOI:** 10.1016/j.redox.2019.101408

**Published:** 2019-12-16

**Authors:** Luciano Ferrada, María Jose Barahona, Katterine Salazar, Peter Vandenabeele, Francisco Nualart

**Affiliations:** a Laboratory of Neurobiology and Stem Cells NeuroCellT, Department of Cellular Biology, Faculty of Biological Sciences, University of Concepcion, Concepcion, Chile; b Center for Advanced Microscopy CMA BIO BIO, University of Concepcion, Concepcion, Chile; c Molecular Signaling and Cell Death Unit, VIB Inflammation Research Center, Ghent, Belgium; d Department of Biomedical Molecular Biology, Ghent University, Ghent, Belgium

**Keywords:** Vitamin C, Ascorbic acid, Dehydroascorbic acid, Necroptosis, Neuronal death, Live cell microscopy

## Abstract

Under physiological conditions, vitamin C is the main antioxidant found in the central nervous system and is found in two states: reduced as ascorbic acid (AA) and oxidized as dehydroascorbic acid (DHA). However, under pathophysiological conditions, AA is oxidized to DHA. The oxidation of AA and subsequent production of DHA in neurons are associated with a decrease in GSH concentrations, alterations in glucose metabolism and neuronal death. To date, the endogenous molecules that act as intrinsic regulators of neuronal necroptosis under conditions of oxidative stress are unknown. Here, we show that treatment with AA regulates the expression of pro- and antiapoptotic genes. Vitamin C also regulates the expression of RIPK1/MLKL, whereas the oxidation of AA in neurons induces morphological alterations consistent with necroptosis and MLKL activation. The activation of necroptosis by AA oxidation in neurons results in bubble formation, loss of membrane integrity, and ultimately, cellular explosion. These data suggest that necroptosis is a target for cell death induced by vitamin C.

## Graphical abstract

**Figure:**
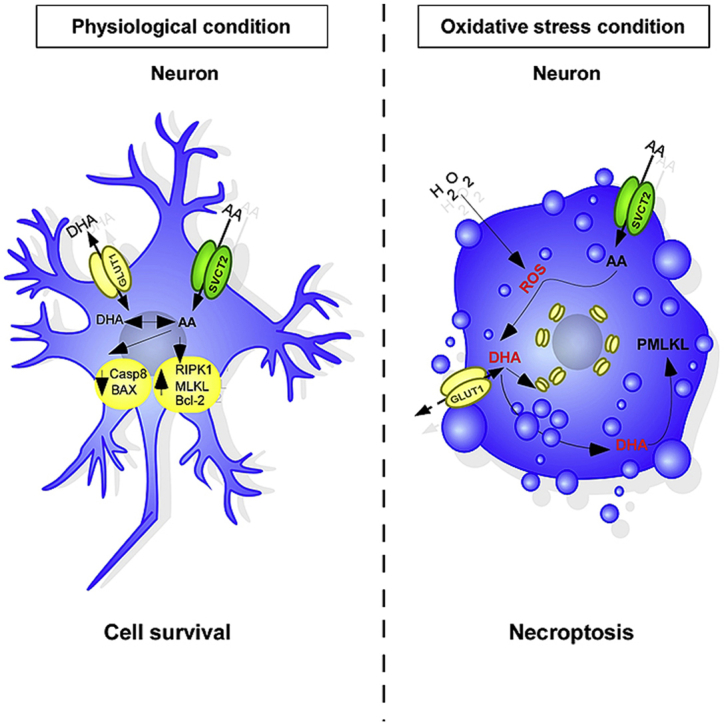

## 1 Introduction

The reduced form of vitamin C, ascorbic acid (AA), is the main antioxidant found in the central nervous system [1]. Under physiological conditions, AA predominates and is taken up by neurons through cotransporter sodium ascorbate 2 (SVCT2) [2]. When vitamin C fulfills its antioxidant function, dehydroascorbic acid (DHA) is produced, which diffuses into nerve cells by GLUTs transporters [3]. However, under cerebral pathophysiological conditions, such as acoustic trauma [4] or ischemia and reperfusion, AA is oxidized to DHA [5,6]. Moreover, patients with Alzheimer's disease have lower plasma concentrations of vitamin C than healthy individuals [7] due to increased oxidative stress [8], which could favor the production and accumulation of DHA.

For many years, it has been thought that vitamin C could act as a pro-oxidant and kill tumor cells [[9], [10], [11]]. In this context, it was recently proposed that DHA could induce cell death directly in KRAS and BRAF mutant colorectal cells [12] and in neuronal cells by triggering metabolic crisis [3,13]. Irrespective of whether AA or DHA is the inducer of cell death, it has been confirmed in the literature that vitamin C mainly induces death independent of caspases with characteristics of necrotic disintegration [9,[14], [15], [16]]. New and emerging forms of regulated cell death with morphological characteristics of necrotic disintegration, including ferroptosis [17,18] and necroptosis [19,20], have been recently reported.

The execution of necroptosis requires that caspase-8 (Casp8) be inhibited or absent [21]. RIPK1 also phosphorylates RIPK3 to activate necroptosis by MLKL (mixed lineage kinase domain-like) phosphorylation [22]. In the case of necroptosis, MLKL undergoes a conformational change following RIPK3-mediated phosphorylation (S345/S347/T349 in mouse and S357/T358 in human) of the autoinhibitory C-terminal pseudokinase domain. This change induces exposure of the toxic N-terminal four helical bundle domain (4HBD), allowing the interaction of positively charged patches with inositalhexaphosphate (IP6) and phopshatidylinositolphosphates (such as PIP, PIP2), followed by insertion and multimerization in the plasma membrane [23]. Then, phosphatidyl serine is exposed, and pores are formed that trigger osmotic stress and death by cell explosion [[24], [25], [26]]. MLKL-dependent cell death by the necroptotic pathway induces the release of DAMP (damage-associated molecular pattern), which promotes inflammation and increases activation of necroptosis and other parallel death pathways [27].

Necroptosis was of particular interest in the current study because this type of death is induced in conditions of cerebral ischemia and reperfusion [20,28,29], Alzheimer's disease [30], and amyotrophic lateral sclerosis (ALS) [31] in which oxidation of AA is favored [[5], [6], [7], [8]]. Cerebral necroptosis is favored over apoptosis because the adult brain is an organ refractory to apoptosis [[32], [33], [34]].

In this study, we determined that the intracellular oxidation of AA favors the subsequent activation of necroptosis by phosphorylation of MLKL. Furthermore, we analyzed the disintegration characteristics in living cells by 4D microscopy, revealing that neuronal cells form extracellular vesicles prior to necroptotic disintegration in a manner similar to that recently reported in the literature [24]. Finally, through CRISPR/Cas9, we generated MLKL^−/-^ and SVCT2^−/−^ neuronal cells and showed that ablation of MLKL delays cell death. Furthermore, if the uptake of AA is inhibited by the deletion of SVCT2, neuronal death is completely prevented. Our results suggest that the oxidation of vitamin C and the generation of DHA could regulate neuronal necroptosis under conditions of oxidative stress.

## 2 Methods

### 2.1 Compounds, antibodies, and reagents

The detailed information on the compounds, antibodies and reagents is listed in Supplementary Table 1. The secondary antibodies included the following: Cy3 AffiniPure Donkey Anti-Goat IgG (H+L) (705-165-147), Alexa Fluor 488 AffiniPure Donkey Anti-Rabbit IgG (H+L) (711-545-152), Cy2 AffiniPure Donkey Anti-Mouse IgG (H+L) (715-225-151), Cy5 AffiniPure Donkey Anti-Rabbit IgG (H+L) (711-175-152), Alexa Fluor 488 AffiniPure Donkey Anti-Rat IgG (H+L) (712-545-153), Cy3 AffiniPure Donkey Anti-Rat IgG (H+L) (712-165-153), peroxidase-AffiniPure Goat Anti-Rabbit IgG (H+L) (111-035-003) and AffiniPure Donkey Anti-Rat IgG (H+L) (712-005-153) were purchased from Jackson ImmunoResearch (West Grove, PA, USA).

### 2.2 Cell lines

Primary cortical neurons were obtained from rat embryos at 17 days gestation as previously described [3]. All the studies were approved by the Animal Ethics Committee of the Chile's National Commission for Scientific and Technological Research (CONICYT, approved protocol #1181243). All animal studies were approved by the Animal Care and Use Committee of the Universidad de Concepción. Animals were treated in compliance with the NIH guidelines for animal care and use. Neuro2a (N2a) cells (CCL-131) were obtained from American Type Cell Collection (ATCC, Rockville, MD, USA) and maintained at 37 °C, 5% v/v CO2 in a humidified incubator with DMEM-F12 (GIBCO) supplemented with 5% FBS (Mediatech, #MD.35-010-CV), 2 mM l-glutamine (GIBCO) and 100 U/mL penicillin-streptomycin (GIBCO). HN33.11 cells were cultured using DMEM-HG (GIBCO) under the same conditions. To ensure mycoplasma-free cultures, the cells were treated with BM cyclin (Roche, #10799050001) prior to experiments. N2a^*(Mlkl−/−)*^ and N2a^*(Svct2−/-)*^ cells were generated by CRISPR/Cas9 using CAG-Cas9-2a-RFP and Cas9-ElecD plasmids (Atum, #pD1321-AP) and transfection with Lipofectamine 3000 (Life Technologies). The gRNA target sequences for the murine initiation codons of MLKL and SVCT2 were GCACACGGTTTCCTAGACGC and TGTAGATCATATCCGACCTC, respectively. The cells were selected at 48 h posttransfection using a BD FACSAria III cell sorter. Single-cell RFP was sorted in 96-well plates. MLKL- and SVCT2-deleted colonies were verified by Western blotting. N2a-hSVCT2wt-EYFP, N2a-EGFP, HN33.11-hSVCT2wt-EYFP, and HN33.11-EGFP cells were generated by infection with lentiviral particles as previously described [2]. Stable EYFP- and EGFP-expressing cells were selected at 72 h postinfection by FACS.

### 2.3 Live-cell microscopy

N2a and HN33.11 cells were seeded in 18-mm cover glasses in 12-well plates for 48 h. After treatment with H_2_O_2_, the cover was removed, and the plates were placed in a live-cell perfusion chamber. Then, the cells were loaded with fluorescent probes for 10 min and washed with PBS. Finally, the cells were incubated in complete medium and imaged at 37 °C and 5% CO_2_ in a confocal spectral Zeiss LSM 780 live-cell system. The images were acquired in 4D (x: 1024, y: 1024, z: 6 or 10, time, channels: 5, 8-bit) with an objective Plan-Apochromat 63x/1.40 Oil DIC M27. The following fluorescent probes were used: Hoechst 33342 (0.1 μg/mL, ex/em (nm) 350/461), Alexa Fluor 488 phalloidin (20 nM, ex/em (nm) 495/518), MitoTracker Red CMXRos (25 nM, ex/em (nm) 579/599), and cellmask (0.3X ex/em (nm) 650/655). Finally, the images were reconstructed in a movie using the Zen lite software (Zeiss).

### 2.4 Immunocytochemistry and image processing

Cells were seeded on coverslips. After treatment, the cells were fixed with 4% paraformaldehyde for 30 min at room temperature, washed with Tris-phosphate buffer [35] and incubated overnight at room temperature with the following antibodies: anti-SVCT2 (1:50), anti-GLUT1 (1:400), anti-RIPK1 (1:400), anti-RIPK3 (1:50), anti-MLKL (1:400), anti-phospho RIPK1 (1:100) and anti-phospho MLKL (1:100). The cells were incubated at room temperature for 2 h with Cy3 AffiniPure Donkey Anti-Goat IgG, Alexa Fluor 488 AffiniPure Donkey Anti-Rabbit IgG, Cy2 AffiniPure Donkey Anti-Mouse IgG, Cy5 AffiniPure Donkey Anti-Rabbit IgG, Alexa Fluor 488 AffiniPure Donkey Anti-Rat IgG or Cy3 AffiniPure Donkey Anti-Rat IgG (1:200). Hoechst 33342 (1:1000) was used for nuclear staining. The images were acquired using an LSM 780 spectral confocal microscope (Zeiss) or ELIRA S.1 Superresolution Structured Illumination Microscopy (Zeiss). The images were exported in .czi format and processed in Imaris v 9.1 software (Bitplane Inc) for 3D reconstruction, colocalization, morphology and bounding box analysis. The intensity profile was determined with ImageJ software.

### 2.5 Cell viability assay

N2a and HN33.11 cells were supplemented with 200 μM AA for 36 h. Then, intracellular oxidation of AA was induced by incubation with 500 μM H_2_O_2_ for 30 min (or the concentration indicated in the figure). After this time, H_2_O_2_ was removed, and the cells were washed with PBS and incubated in complete medium for 3 h. Finally, cell viability was measured by XTT (Biological Industries #20-300-1000) colorimetric analysis. Cell death by loss of plasma membrane integrity was measured by flow cytometry (BD FACSAria III) with 500 nM TOPRO-3 (10 min) [36]. The flow cytometry data were processed with FlowJo software (Tree Star). Nec-1, Nec-1s and zVAD.FMK were used during and after treatment with H_2_O_2_.

### 2.6 Measurement of ROS

The cells were trypsinized, resuspended in serum-free DMEM-F12 (GIBCO), incubated for 30 min with 500 nM CellROX Deep Red (Life Technologies) and analyzed by flow cytometry (BD FACSAria III). The flow cytometry data were processed with FlowJo software (Tree Star).

### 2.7 Intracellular measurement of AA

The cells were washed with PBS, trypsinized and resuspended in cold PBS. AA was measured using the ferric reducing (antioxidant) activity and ascorbic acid concentration (FRASC) colorimetric assay (bioassay system #EASC-100) according to the manufacturer's instructions.

### 2.8 Western blot analysis

N2a and HN33.11 cells were lysed with NP-40 buffer supplemented with a protease/phosphatase inhibitor cocktail (Cell Signaling #5872). The proteins (30–50 μg) were separated using 10% SDS-PAGE gel, transferred to PVDF membranes (0.45-μm pore; Immobilon-P #IPVH00010, Merck Millipore) and probed with anti-SVCT2 (1:1000), anti-RIPK1 (1:3000) and anti-MLKL (1:5000) overnight. The membranes were then incubated with secondary antibodies, including HRP-conjugated donkey anti-goat IgG, donkey anti-rabbit IgG, donkey anti-rat IgG (1:6000) and anti-actin-HRP (1:50000) for 2 h at room temperature. The reaction was developed using the Western Lighting® Plus-ECL enhanced chemiluminescence substrate (PerkinElmer, Waltham, MA, USA).

### 2.9 Quantification of proteins by flow cytometry

N2a and HN33.11 cells were trypsinized and fixed in 4% PFA at room temperature for 10 min. Then, the cells were permeabilized in 100% cold methanol for 30 min. The cells were washed twice by centrifugation in incubation buffer (0.5% BSA in Tris-phosphate buffer). For immunostaining and resuspended in 100 μL of incubation buffer with diluted RIPK1 (1:100) and MLKL (1:100) primary antibodies. After 1 h, the cells were washed by centrifugation and resuspended in Alexa Fluor 488 AffiniPure Donkey Anti-Rabbit IgG (1:200) or Alexa Fluor 488 AffiniPure Donkey Anti-Rat IgG (1:200) secondary antibodies for 30 min. Finally, the cells were washed in incubation buffer, resuspended in PBS and analyzed by flow cytometry. The flow cytometry data were processed with FlowJo software (Tree Star).

### 2.10 Real-time PCR

Total RNA from N2a and HN33.11 cells was purified using Trizol reagent (Invitrogen, #15596018). Real-time PCR was performed with Brilliant II SYBR® Green QPCR Master Mix (Agilent Technologies, #600828). PCR (95 °C for 10 min; 95 °C for 30 s; 60 °C for 20 s; 72 °C for 20 s; 40 cycles) was carried out in Mastercycler Realplex^2^ (Eppendorf). The primers used are listed in Supplementary Table 2. The relative expression of mRNA to cyclophilin was calculated using the 2^−ΔΔCt^ method.

### 2.11 Statistical analysis

Statistical analyses were performed using GraphPad Prism version 6.01. In the legend of each figure, the exact values of *n* of each analysis are described. In all cases, two-tailed Student's t-tests were used. *p-*values ≤0.05 were considered statistically significant. Data are presented as the means ± SEM.

## 3 Results

### 3.1 Intracellular AA oxidation induces neuronal death, balloon-like morphology and subcellular redistribution of vitamin C transporters

**Fig. 1:**
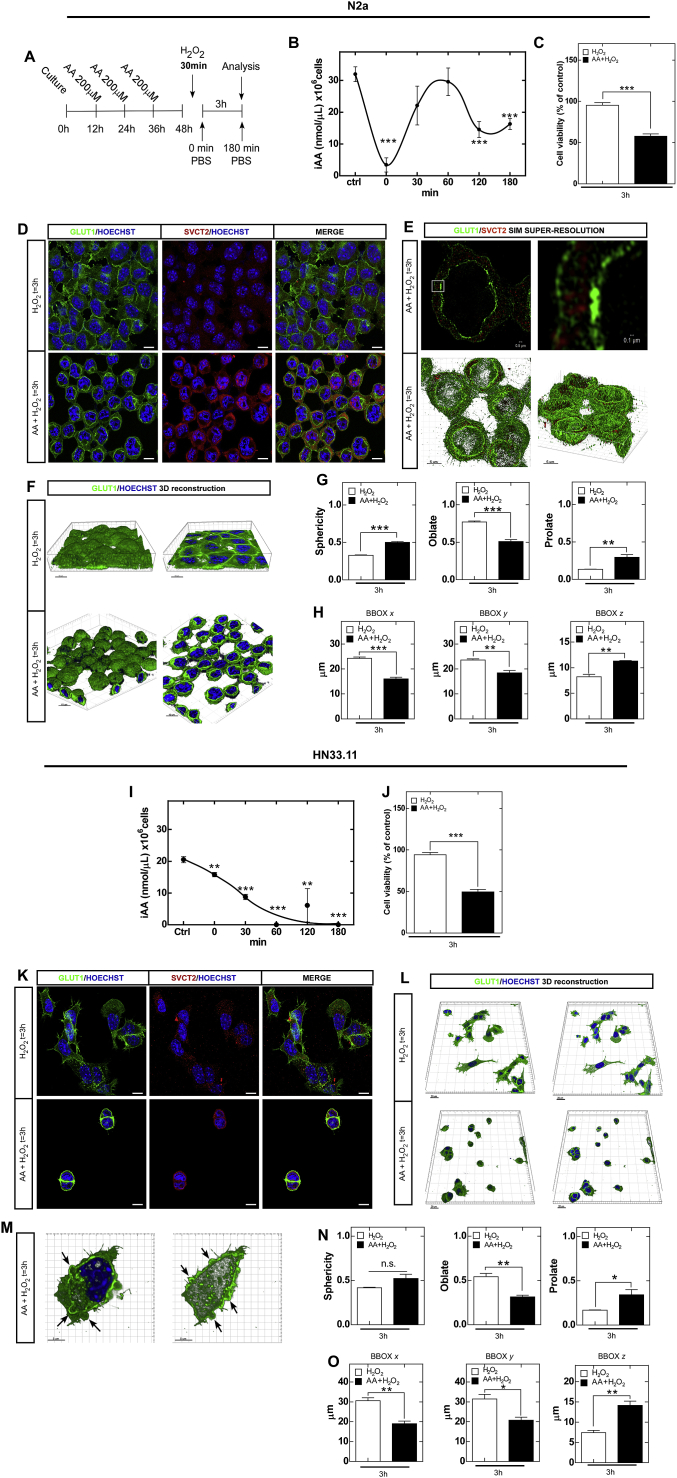
Oxidation of vitamin C induced neuronal death and morphological changes. (A) Schematic of the treatment protocol with vitamin C and oxidative stress induction. (B and I) Intracellular measurement of AA. The control condition represents the intracellular concentration of AA prior to treatment with H_2_O_2_. The time corresponds to the time in minutes posttreatment with H_2_O_2_. n = 3 biologically independent samples. (C and J) Cell viability at 3 h posttreatment. (D and K) GLUT1 and SVCT2 distribution at 3 h posttreatment. (E) 2D and 3D superresolution SIM of N2a cells at 3 h posttreatment. (F and L) Imaris 3D reconstruction at 3 h posttreatment. (G and N) Morphological analysis in N2a and HN33.11 cells. (H and O) Cell size analysis with the Imaris bounding box tool. (M) 3D morphological alterations of the plasma membrane in HN33.11 cells; the arrows show bubble-like structures. Scale bar 10 μm. Data are shown as the mean ± SEM (two-tail Student's t-tests); n = 3 biologically independent samples. All data are representative of three separate experiments. *p ≤ 0.05, **p ≤ 0.01, ***p ≤ 0.001.

To simulate the physiological brain conditions during which neuronal cells accumulate AA [1,37], we supplemented *in vitro* cultures of the neuronal N2a and HN33.11 cell lines and cortical neurons with 200 μM AA for 36 h, as shown in Fig. 1A. To favor the intracellular oxidation of AA, we induced oxidative stress by glucose deprivation and temporary addition of 0.5 mM H_2_O_2_ in N2a and HN33.11 cells and 50 μM H_2_O_2_ in cortical neurons for 30 min, after which the H_2_O_2_ was removed. The cells were left in complete medium for 3 h to perform the analyses (Fig. 1A). Using the FRASC method, we found that the cell lines accumulated AA and that H_2_O_2_ treatment induced intracellular AA oxidation over time (Fig. 1B and I). Furthermore, viability analyses with XTT showed that oxidation of AA induced 50% neuronal death at 3 h posttreatment in both cell lines and in cortical neurons (Fig. 1C and J, S1F), whereas treatment with AA alone had no effect on cell viability (Fig. S1A). Because the reduced form of vitamin C, AA, is transported by SVCT2 in the brain and the transport of the oxidized form, DHA, is mediated by GLUTs [1,38], we analyzed the presence of these transporters by means of spectral confocal microscopy and superresolution structured illumination microscopy (SIM). Our results showed that both neuronal cell lines and cortical neurons expressed vitamin C transporters (Fig. 1D and K, S1G). However, when AA oxidation was induced in N2a cells, subcellular vitamin C transporter redistribution was observed: GLUT1 was detected in the perinuclear compartment, while SVCT2 was primarily observed near the plasma membrane (Fig. 1D).

It has been reported that the morphological characteristics of necroptosis in peripheral cells include a balloon-like morphology [26]. In addition to inducing a change in the distribution of GLUT1/SVCT2 vitamin C transporters, we observed that the oxidation of vitamin C induced a drastic "balloon-like" morphological change in neuronal cells, as revealed by 3D reconstruction using GLUT1 labeling (Fig. 1F, L, Supplementary Videos 1 and 2). This change in cell morphology was accompanied by the formation of bubble-like structures (Fig. 1M), a morphological characteristic of cell death due to necroptosis [24]. Using live-cell microscopy and 3D reconstruction (Imaris v 9.1 software, Bitplane Inc), we confirmed that treatment with TNF and zVAD also induced a balloon-like morphology in neuronal cells (Figs. S1B and S1E, Supplementary Video 3, video 4). On the other hand, 3D reconstruction analysis showed a perinuclear structure generated by GLUT1 in the whole cell (Fig. 1E and F). To study this internal GLUT1 double-membrane structure in further detail, we analyzed GLUT1 with superresolution SIM in 2D and 3D. Our data showed that the approximate thickness of this double membrane structure is 100 nm and that in the intermembrane space contains "trapped" SVCT2 (Fig. 1E). This perinuclear double-membrane structure does not correspond to the nuclear membrane because it does not colocalize with the nuclear lamina (Fig. S1C). In addition, we did not find increased expression of SVCT2 or GLUT1 by qRT-PCR analysis (Fig. S1D). To examine cell size changes, we used the bounding box tool, which identifies an object by considering the minimal rectangular box that fully encloses the object, whose faces are aligned parallel to the axes of a coordinate system. Using this method, we observed that the oxidation of AA induces a drastic change in cell size; in both cell lines, the cell lengths decreased along the x and y axes, while the cell increased in length along the z axis (Fig. 1H, O). The analysis of cell size and shape are closely related because the loss of normal morphology (oblate, a spheroid with flattened poles) is explained by the shortening of the *x* and *y* axes. The spherical and prolate (an elongated spheroid) morphology is explained by the increase in the length of the *z* axis (Fig. 1G, H, N, O).

Supplementary video related to this article can be found at https://doi.org/10.1016/j.redox.2019.101408.

The following are the supplementary data related to this article:

Multimedia component 3 Multimedia component 3

Multimedia component 4 Multimedia component 4

Multimedia component 5 Multimedia component 5

Multimedia component 6 Multimedia component 6

### 3.2 Vitamin C oxidation induces cell death by a mechanism independent of ROS and apoptosis

**Fig. 2:**
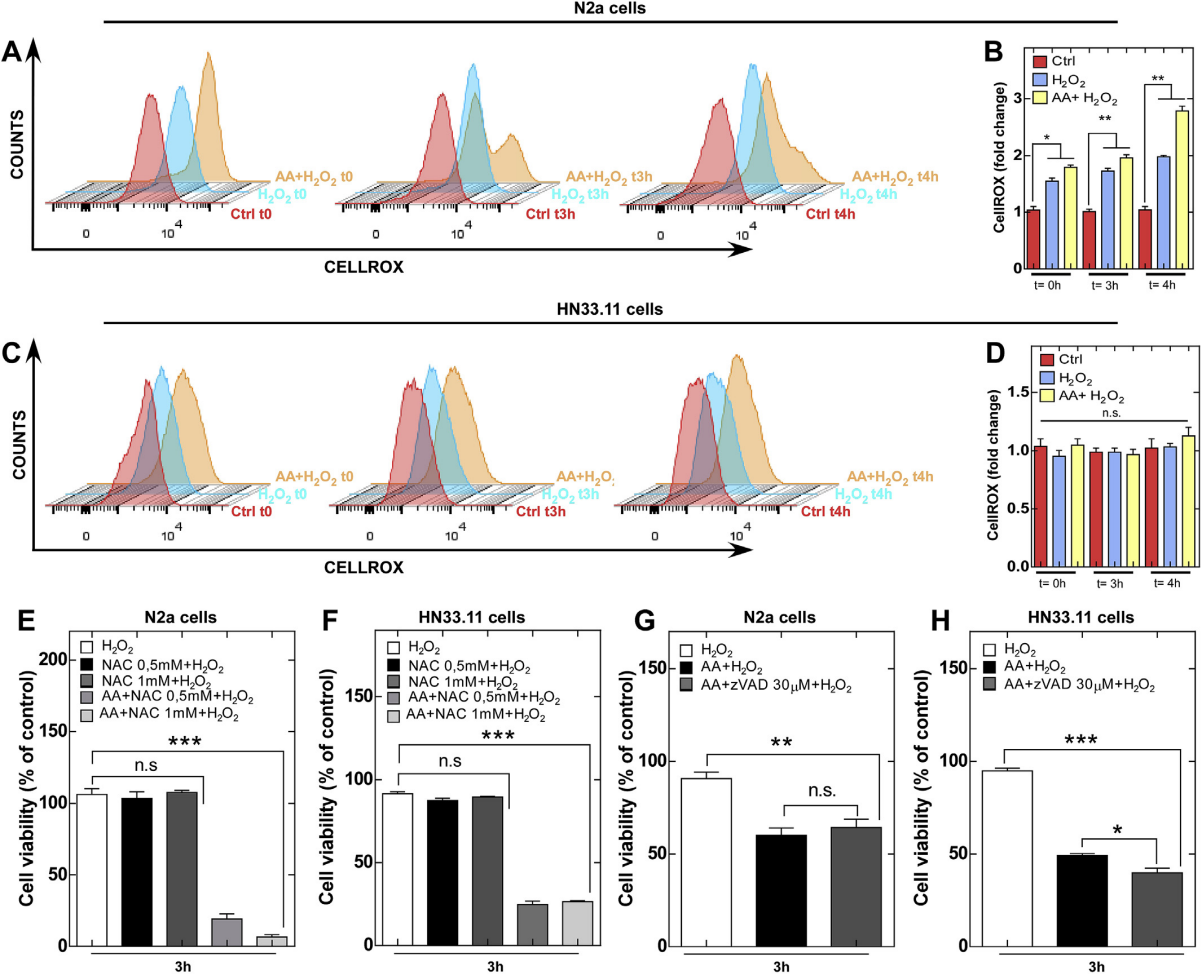
ROS and apoptosis inhibition do not prevent cell death induced by vitamin C oxidation.(A and C) Intracellular ROS production in N2a and HN33.11 cells. (B and D) Quantification of ROS production with respect to control (n = 2 biologically independent samples). (E and F) Cell viability analysis of ROS inhibition with N-acetyl-cysteine (NAC, 24 h before treatment) in N2a and HN33.11 cells (n = 3 biologically independent samples). (G and H) Cell viability analysis of apoptosis inhibition in N2a cells (n = 3 biologically independent samples). Data are shown as the mean ± SEM (two-tail Student's t-tests); all data are representative of three separate experiments. *p ≤ 0.05, **p ≤ 0.01, ***p ≤ 0.001, n.s., not significant.

The activation of necroptosis has been related to increased ROS production in certain peripheral cell lines [26]. Thus, we evaluated the production of ROS at different times by means of flow cytometry and the CellROX Deep Red probe, which detects total cytoplasmic ROS. Our results showed that the cell lines analyzed exhibited differential ROS production. In N2a cells, H_2_O_2_ induced intracellular ROS production at time 0 posttreatment (blue peak) (Fig. 2A and B).

As expected, at 3 and 4 h after treatment, the cells that were treated with H_2_O_2_ exhibited increased ROS levels over time (blue peak) (Fig. 2A and B). Unexpectedly, the cells that previously accumulated AA exhibited higher levels of ROS (orange peak) (Fig. 2A and B). In N2a cells in which AA had previously accumulated, we observed that two cell populations were generated [1]: a cell population with low ROS levels, probably due to the antioxidant effect of AA, and [2] a cell population with high levels of ROS (orange peak) (Fig. 2A and B), which could eventually correspond to cells in the process of cell death. In HN33.11 cells, we found that H_2_O_2_ or H_2_O_2_ + AA treatment did not generate an increase in ROS production over time (Fig. 2C and D), which could suggest that depending on the neuronal type, there are different antioxidant mechanisms not necessarily related to vitamin C. To determine if the production of ROS participates in the death-inducing mechanism regulated by vitamin C, we inhibited ROS production using the antioxidant N-acetyl-cysteine (NAC). However, our results show that inhibition of ROS by NAC does not prevent neuronal death under oxidative conditions of vitamin C (Fig. 2E and F), suggesting that the mechanism of death is independent of ROS and the antioxidant function of vitamin C. Finally, an effective dose of pan-caspase inhibitor, zVAD.FMK (Figs. S2A and S2B) did not protect against vitamin C oxidation-mediated cell death (Fig. 2G and H), suggesting a nonapoptotic mechanism. Thus, there are likely differential mechanisms of activation of nonapoptotic cell death in the brain that do not necessarily involve increased ROS production.

### 3.3 Supplementation with AA alters the expression of RIPK1, RIPK3 and MLKL, favoring necroptosis under conditions of oxidative stress

**Fig. 3:**
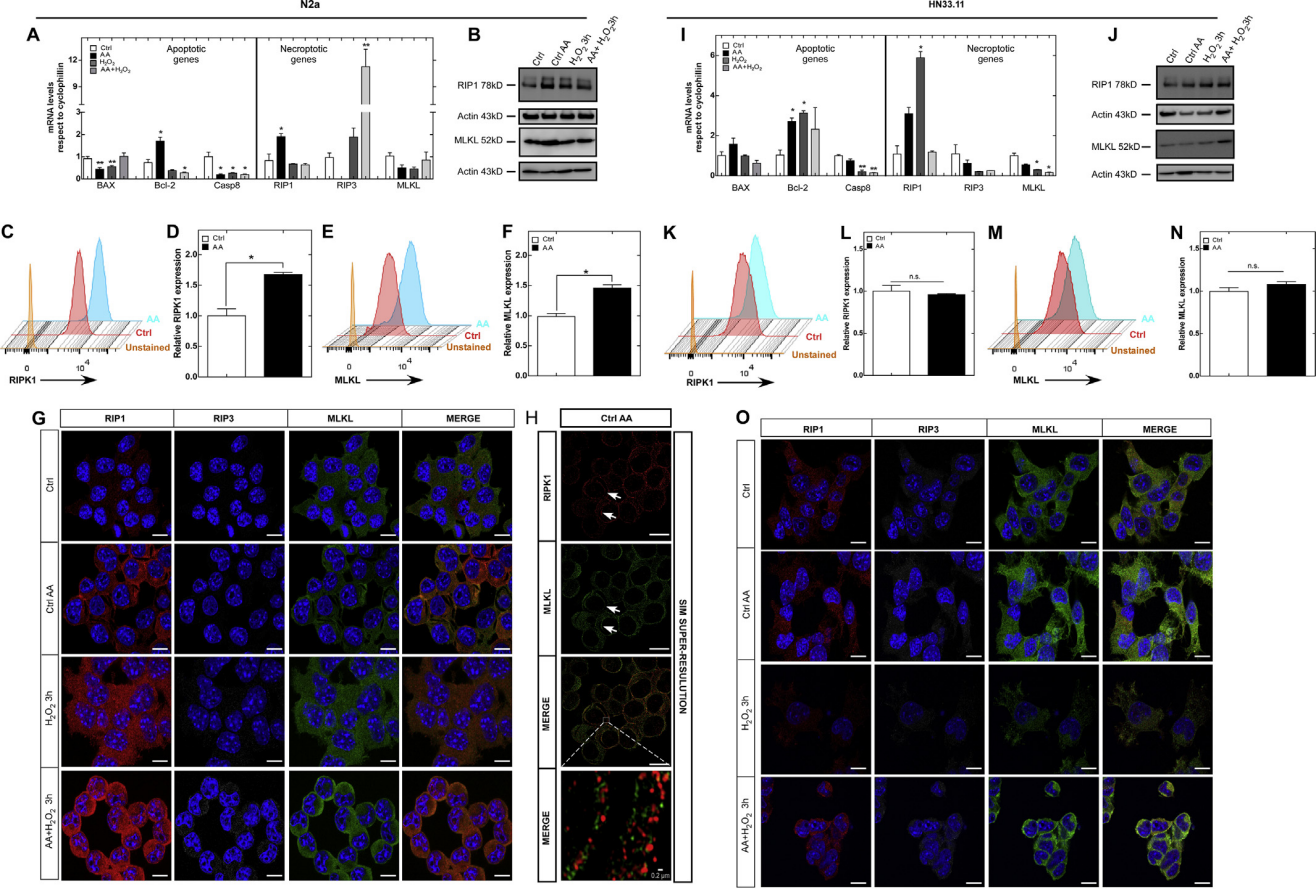
Vitamin C regulates the expression of RIPK1, RIPK3 and MLKL, stimulating necroptosis. (A and I) mRNA levels of apoptotic and necroptotic genes. (B and J) Expression of RIPK1 and MLKL in N2a and HN33.11 cells. (C and K) RIPK1 expression determined by flow cytometry (50,000 counts). (D and L) Relative quantification of RIPK1 by flow cytometry (n = 2 biologically independent samples). (E and M) MLKL levels determined by flow cytometry (50,000 counts). (F and N) Relative quantification of MLKL by flow cytometry (n = 2 biologically independent samples). (G and O) Analysis of the expression and localization of RIPK1, RIPK3 and MLKL. (H). SIM superresolution of RIPK1 and MLKL in N2a cells treated with AA. Scale bar 10 μm. Data are shown as the mean ± SEM (two-tail Student's t-tests). n = 3 biologically independent samples. The experiment was independently reproduced three times. *p ≤ 0.05, **p ≤ 0.01, ***p ≤ 0.001, n.s. not significant.

AA is a cofactor of demethylases that regulates the expression of different genes [39]. To examine whether nonapoptotic necrotic cell death could involve an alternative mechanism of necroptosis induction, we determined whether treatment with AA or the oxidation of AA could affect the expression levels of apoptotic and necroptotic factors. In a first approach, we evaluated the mRNA levels of apoptotic genes (bcl-2, bax and casp8) and necroptotic genes (ripk1, ripk3, mlkl) by qRT-PCR. Similar to previous studies [[40], [41], [42]], AA supplementation favored inhibition of apoptosis by overexpression of bcl-2 and suppression of bax and casp8 mRNA expression (Fig. 3A and I). However, in cortical neurons, treatment with AA did not alter the mRNA expression of bcl-2, bax or casp8 (Fig. S3A). In contrast, intracellular accumulation of AA favored a significant increase in the expression of RIPK1 mRNA (Fig. 3A and I), whereas in cortical neurons, a significant increase in RIPK3 mRNA was observed (Fig. S3A). Interestingly, in the N2a cell line, ripk3 mRNA was undetectable after AA treatment. With vitamin C oxidation, RIPK3 mRNA expression increased by approximately 12-fold in N2a cells and 3-fold in cortical neurons compared with the control condition (Figs. 3A and S3A). At the protein level, we found that supplementation with AA favored the expression of RIPK1 and MLKL (Fig. 3B and J). Furthermore, quantification by flow cytometry showed that AA induced RIPK1 protein expression by 70% (Fig. 3C and D), while MLKL expression increased by 50% in N2a cells (Fig. 3E and F). However, the levels of RIPK1 and MLKL did not show significant changes in HN33.11 cells (Fig. 3K–N).

We also analyzed the expression and intracellular distribution of RIPK1, RIPK3 and MLKL proteins by confocal spectral microscopy. Our immunocytochemical results maintained the same pattern of expression described above at the mRNA level. However, redistribution to the plasma membrane and the formation of filamentous structures of RIPK1 and MLKL were observed in the N2a cell line compared with AA supplementation (Figs. 3G, H, S3D). This finding suggested that AA controls other signals in addition to RIPK1 and MLKL expression that affect their distribution. However, this distribution pattern was not observed in HN33.11 cells or in cortical neurons (Fig. 3O, S3B). Conversely, H_2_O_2_ treatment did not alter the distribution of necroptotic proteins. In N2a cells, H_2_O_2_ favored the expression of RIPK1 and MLKL (Fig. 3G), whereas the opposite effect was observed in HN33.11 cells (Fig. 3O). However, we observed that both cell lines responded to vitamin C oxidation in a similar fashion, losing their processes, acquiring a "balloon-like" morphology and increasing the expression of RIPK1, RIPK3 and MLKL (Fig. 3G, O). In addition, RIPK1 and MLKL were colocalized in the plasma membrane (Fig. S3F), suggesting activation of the necroptotic pathway by necrosome formation (Fig. 3G, O) and the bubble-like structure containing MLKL and RIPK1 (Fig. S3E).

### 3.4 The oxidation of vitamin C induces neuronal necroptosis

**Fig. 4:**
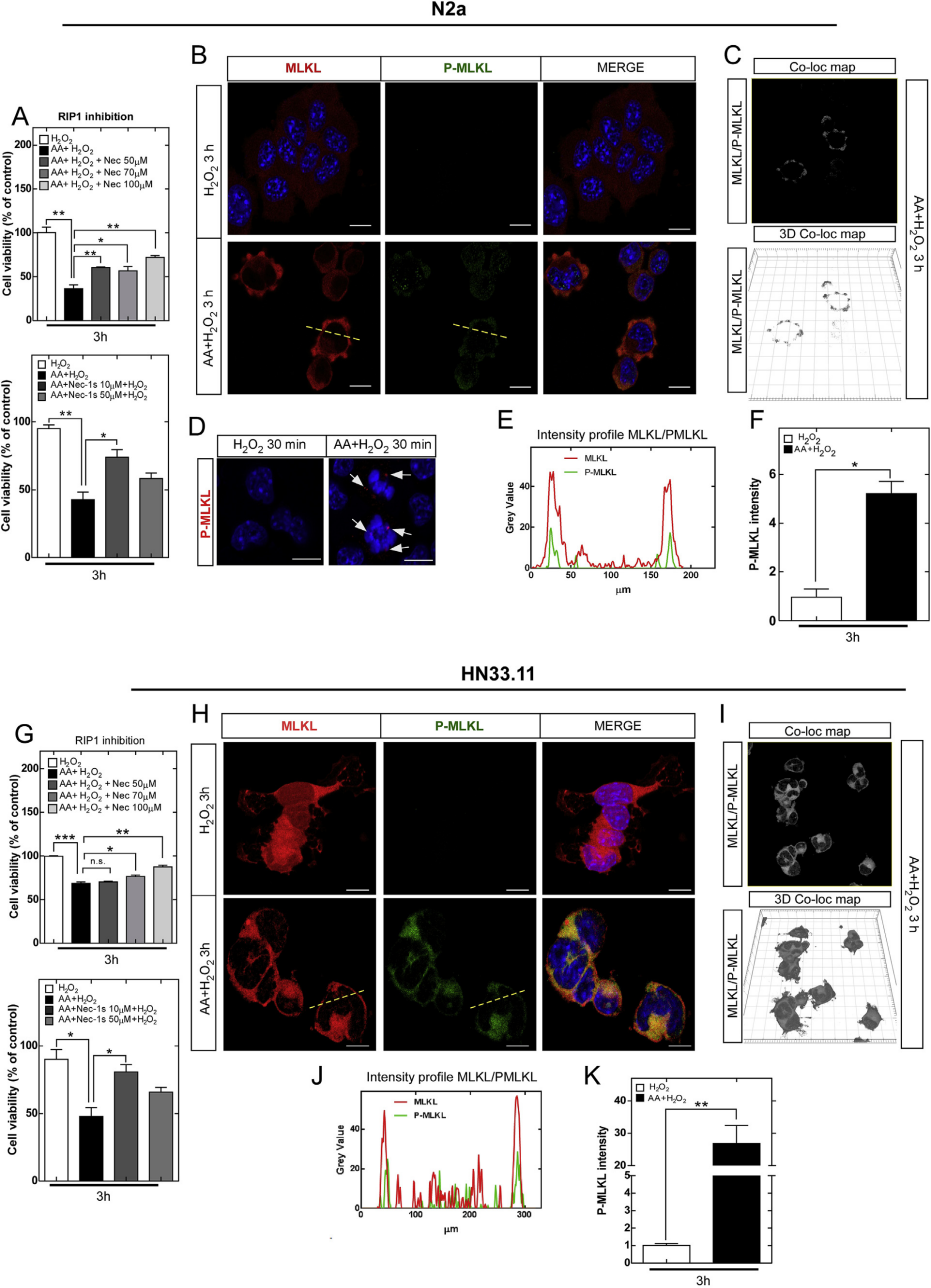
Vitamin C oxidation induces neuronal necroptosis. (A and G) Necroptosis inhibition with Nec-1 or Nec-1s. (B and H) Microphotographs analyses of MLKL phosphorylation. (C and I) Colocation map of MLKL/P-MLKL determined with Imaris. (D) Nuclear translocation of MLKL by phosphorylation. (E and J) Intensity profile of MLKL/P-MLKL. (F and K) Quantification of MLKL phosphorylation. Scale bar 10 μm. Data are shown as the mean ± SEM (two-tail Student's t-tests). n = 3 biologically independent samples. The experiment was independently reproduced three times. *p ≤ 0.05, **p ≤ 0.01, ***p ≤ 0.001, n.s. not significant.

Because AA oxidation induces neuronal death with a morphology different from apoptosis, by a mechanism independent of caspases, and by altering the expression of necroptotic genes and proteins, we analyzed this pathway using necrostatin-1 (Nec-1) or necrostatin-1s (Nec-1s), an inhibitor of RIPK1 kinase activity. Interestingly, both Nec-1 and Nec-1s prevented neuronal death induced by AA oxidation in a dose-dependent manner (Fig. 4A and G, S3C). We report that high concentrations of Nec-1s can be used in primary cultures of cortical neurons (Fig. S3C). In contrast, high concentrations of Nec-1 or Nec-1s show only partial protection or toxicity in neuronal cell lines (Fig. 4A and G, S3C), similar to results described by other groups [43,44]. The execution of necroptosis was supported by the immunocytochemistry finding of MLKL phosphorylation. Our data suggest that AA oxidation induces phosphorylation and rapid MLKL nuclear translocation (Fig. 4B, D, 4H) as well as cocolocalization of MLKL and P-MLKL at the plasma membrane (Fig. 4C and I) and in the whole cell (Fig. 4E and J). Interestingly, we found that MLKL phosphorylation was more intense in HN33.11 cells than in N2a cells (Fig. 4F and K).

### 3.5 Neuronal death induced by AA oxidation shares the characteristics of necroptotic disintegration

**Fig. 5:**
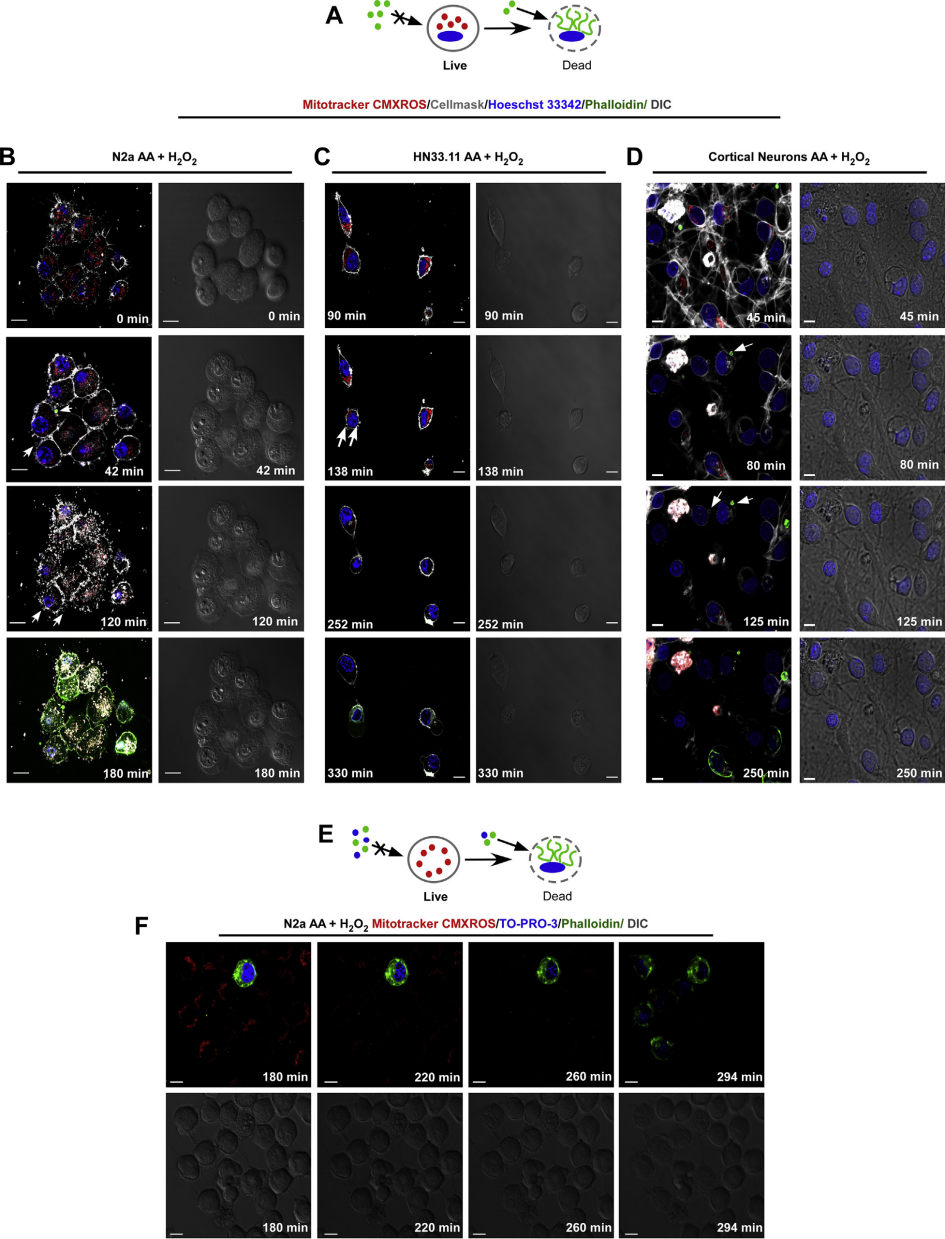
Neuronal vitamin C oxidation induces necroptotic disintegration features. (A and E) Schematic of the live/dead discrimination process. (B, C and D) Bubble formation and features of disintegration during neuronal necroptosis. (F) Shedding of the cytoplasm and features of disintegration during neuronal necroptosis. TOPRO-3 and phalloidin Alexa-488 were used as indicators of cell death by measuring plasma membrane integrity. Scale bar, 10 μm, for cortical neurons, 5 μm.

Necroptosis has been morphologically characterized in peripheral cells by induction of a balloon-like morphology, loss of mitochondrial activity [26] and formation of bubbles in the plasma membrane [24], triggering cellular explosion. Because AA oxidation induces neuronal death due to RIPK1 kinase-mediated necroptosis, we evaluated the characteristics of neuronal disintegration (Fig. 5A and E) by 4D live-cell microscopy (*x, y, z* and time). N2a, HN33.11 cells and cortical neurons maintained nuclear integrity, lost mitochondrial activity as measured by MitotrackerCMXRos stain and formed bubbles permeable to phalloidin prior to the loss of plasma membrane integrity (Fig. 5B, C, 5D Supplementary Videos 5, 6, 7), confirming that the released vesicles corresponded to damaged plasma membrane. It should be noted that the process of cell death and the formation of bubbles (Fig. 5B) were faster in N2a cells than in HN33.11 cells (Fig. 5C) and cortical neurons (Fig. 5D). However, both cell lines converged in that all cells lost plasma membrane integrity, as observed by extending the imaging time (Fig. 5C and F, Supplementary Video 8). Because the live image cell analysis revealed a loss of mitochondrial membrane potential (MitotrackerCMXRos stain [45]), we confirmed that there was fragmentation and alterations in the mitochondrial morphology in response to the oxidation of vitamin C in N2a cells and cortical neurons (Figs. S4A–H, S5A-J). However, inhibition of Drp-1 with Mdivi-1 did not prevent neuronal death (Fig. S4J, S5L). Therefore, Drp-1 does not participate in the neuronal necroptotic pathway as described in peripheral cells [46,47].

Supplementary video related to this article can be found at https://doi.org/10.1016/j.redox.2019.101408.

The following are the supplementary data related to this article:

Multimedia component 7 Multimedia component 7

Multimedia component 8 Multimedia component 8

Multimedia component 9 Multimedia component 9

Multimedia component 10 Multimedia component 10

### 3.6 Overexpression of SVCT2 induces neuronal death in response to oxidation of vitamin C, whereas MLKL^−/-^ or SVCT2 ^−/−^ cells are insensitive to neuronal death induced by vitamin C oxidation

**Fig. 6:**
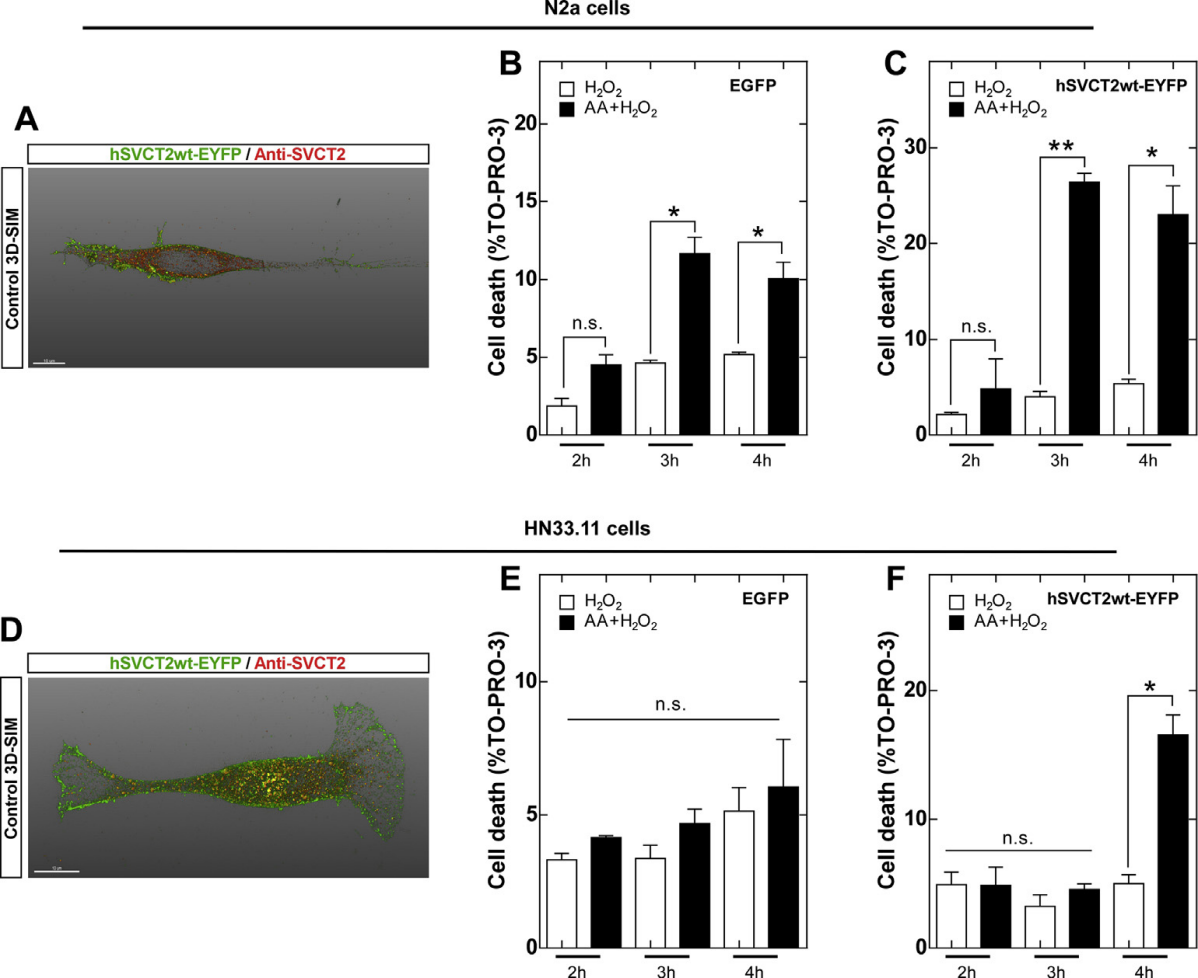
Overexpression of hSVCT2 increases neuronal death induced by vitamin C oxidation. (A and D) SIM superresolution of overexpression of hSVCT2. (B and E) Quantification of cell death in stable N2a and HN33.11 cells transduced with control EGFP (30,000 count). (C and F) Quantification of cell death in stable N2a and HN33.11 cells transduced with hSVCT2wt-EYFP (30,000 count). Scale bar, 10 μm. Data are shown as the mean ± SEM (two-tailed Student's t-tests). n = at least two independent biological replicates. The experiment was independently reproduced three times. *p ≤ 0.05, **p ≤ 0.01, ***p ≤ 0.001, n.s. not significant.

The current data suggest that AA oxidation induces necroptosis. To examine whether this effect is due to the uptake, accumulation and subsequent oxidation of AA, we generated cells that stably overexpressed the human vitamin C transporter, hSVCT2, by means of lentiviral infection and subsequent selection by FACS (Fig. 6A and D). Overexpression of hSVCT2 in neuronal cells greatly increased neuronal death under oxidative conditions induced by H_2_O_2_ (Fig. 6C and F) compared with the control cells, which were infected with a virus that only expresses EGFP (Fig. 6B and E). Furthermore, we determined that the effect of cell death is more accelerated in N2a cells (Fig. 6C) than in HN33.11 cells (Fig. 6F).

**Fig. 7:**
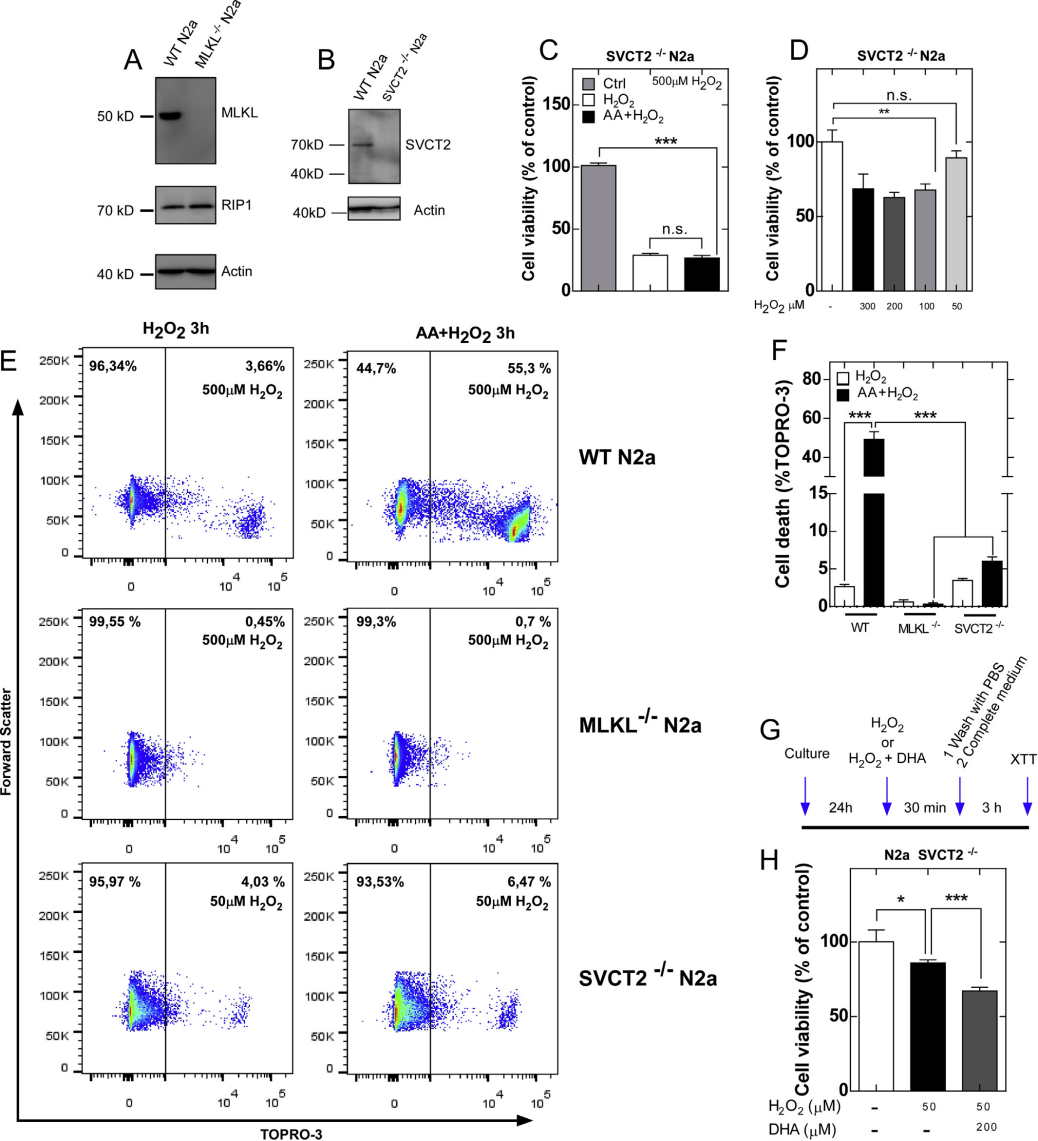
MLKL or SVCT2 KO prevents cell death induced by vitamin C oxidation. (A and B) CRISPR/cas9 validation for MLKL and SVCT2. (C and D) Cell viability at 3 h posttreatment in SVCT2^−/−^ N2a cells. (E and F) Quantification of cell death in WT, MLKL^−/-^ and SVCT2^−/−^ N2a cells. (G) Schematic of the protocol used in Fig. H. (H) Cell viability at 3 h posttreatment in SVCT2^−/−^ N2a cells. Data are shown as the mean ± SEM (two-tail Student's t-tests). n = 3 biologically independent samples. Each experiment was independently reproduced three times. *p ≤ 0.05, **p ≤ 0.01, ***p ≤ 0.001, n.s. not significant.

Because the depletion of MLKL has been used to evaluate the involvement of necroptosis in various models [24], we generated MLKL^−/-^ N2a cells through CRISPR/cas9 (Fig. 7A). The expression of other components of the necroptotic pathway, such as RIPK1, was not altered in MLKL^−/-^ cells (Fig. 7A). Next, we analyzed whether the depletion of MLKL prevents neuronal death induced by vitamin C. As shown in Fig. 7E and F, neuronal death induced by the oxidation of vitamin C was absent in MLKL^−/-^ cells. Live-cell microscopy confirmed the absence of morphological changes or plasma membrane permeability in MLKL^−/-^ cells at 3 h after treatment (Fig. S6A). Because other research groups have shown that blockade of MLKL expression only delays cell death in necroptotic conditions [48], we also analyzed cell death in MLKL^−/-^ cells at 6 h posttreatment and found increased cell death independent of caspases, as the addition of zVAD did not prevent cell death (Fig. S6B). Interestingly, when MLKL^−/-^ cells were treated with Nec-1, cell death was still observed at 6 h (Fig. S6B), suggesting that MLKL-independent cell death is not mediated by RIPK1 kinase activity.

To analyze the effect of AA uptake inhibition, we generated SVCT2^−/−^ N2a cells by CRISPR/cas9 (Fig. 7B). Using the FRASC method, we did not detect intracellular AA after three supplementations with 200 μM AA every 12 h (data not shown). Using the previously described oxidative stress induction protocol (Fig. 1A), neuronal death induced by vitamin C oxidation was completely prevented (Fig. 7C). At the same time, SVCT2^−/−^ cells were highly sensitive to oxidative stress induced by 0.5 mM H_2_O_2_ (Fig. 7C), which was not the case in wild-type cells.

Following a dose response assay with decreasing concentrations of H_2_O_2_ (Fig. 7D), we selected 50 μM H_2_O_2_ for subsequent experiments to reach death rates similar to wild-type cells. When SVCT2^−/−^ N2a cells were supplemented with AA to induce oxidative stress, cell death was completely prevented (Fig. 7E and F), suggesting that cell death is dependent on an intracellular mechanism of vitamin C accumulation and oxidation. Interestingly, when SVCT2^−/−^ N2a cells were subjected to oxidative stress in the presence of DHA, which is incorporated into cells through GLUTs but independent of SVCT2, as described in Fig. 7G, neuronal death was induced (Fig. 7H). Thus, our data suggest that intracellular AA is required for additional sensitization and eventual necroptosis. At the same time, suggest that DHA is the molecule that induces lethality in neurons.

## 4 Discussion

There are different data indicating that AA depletion (due to its oxidation to DHA) could affect the activity of enzymes important for the function of specific neurons in the CNS [38]. Among them, dopamine β-Hydroxylase, which is essential in the synthesis of epinephrine, as well as prolyl 4-hydroxylase (three isoenzymes) that participates in the hydroxylation of proline residues in hypoxia-inducible factor (HIF), could be affected. Dysfunction of these enzymes can change neuronal metabolism, inducing cell death [14]. In this work, we have analyzed how oxidized vitamin C affects neuronal survival in conditions of oxidative stress, inducing non-apoptotic cell death.

It has long been assumed that "vitamin C" (without discriminating between AA and DHA redox states) can act as a pro-oxidant and induce cell death due to oxidative stress associated with a Fenton reaction and extracellular production of H_2_O_2_ [9,10], thereby inducing oxidative necrotic cell death. However, our results suggest that there is an intracellular mechanism of signal transduction regulated specifically by the oxidized form of vitamin C, DHA, leading to necroptotic cell death. The morphological changes induced by the oxidation of vitamin C from our experiments in neurons resemble those that occur in peripheral cells in response to TNF in the presence of the pancaspase inhibitor zVAD-fmk [24,26]. However, it should be noted that in our experimental conditions, the pathway of necroptosis does not require death ligands. Thus, DHA could function as an intrinsic activator of necroptosis. Furthermore, although several antioxidants have been used to prevent cell death, our model suggests that the antioxidant function of AA does not impact neuronal necroptosis because inhibition of ROS by NAC does not prevent neuronal death, which is similar to the results described by other research groups [20,29]. Interestingly, supplementation of neurons with AA increased the expression and redistribution (filamentous structures) of RIPK1 and MLKL in the absence of oxidative stress. Regarding the increased expression of RIPK1 and its redistribution, increased *p*-ERK levels promote the expression of RIPK1, which may be associated with inhibition of apoptosis [49], while its localization in the plasma membrane could correspond to ubiquitination [50]. Interestingly, previous results from our laboratory have shown that AA is a potent inducer of ERK phosphorylation in neuronal cells [2]. Thus, AA may activate ERK, thereby promoting RIPK1 expression and inhibiting death by apoptosis. Although little is known about the mechanisms that control the expression of MLKL, our data suggest that increased levels of MLKL may not be directly related to death processes given that AA supplementation in the absence of oxidative stress does not induce neuronal death (Fig. S1A). It is possible that increased MLKL expression could be related to nonnecroptotic functions, such as receptor-mediated endocytosis and exosome production [51,52]. Deletion of MLKL only delayed but did not prevent cell death in our study. In this context, the deletion of MLKL in the neuron leads to a change from neuronal death dependent on RIPK1-MLKL to RIPK1- and MLKL-independent neuronal death. We speculate that the accumulation of DHA in the absence of MLKL could cause metabolic crisis given that DHA is a potent inhibitor of hexokinase [53] and a glycolysis inhibitor in neurons [3]. Thus, the accumulation of DHA in cells deficient in MLKL could generate a change from necroptosis to other nonapoptotic types of death, such as autosis, because DHA can also act as an autophagy stimulator [54]. Thus, MLKL would not be the best target for the prevention of neuronal death under pathophysiological conditions. Previous studies have determined that DHA blocks the activation of NFκB because it is a potent and specific inhibitor of IKKα/β and p38 kinase activity in response to TNF-α [55,56]. Our results suggest for the first time that the oxidation of AA to DHA results in neuronal death due to the activation of necroptosis. We propose that the regulatory mechanism would be through RIPK1 because its activity can be inhibited by phosphorylation dependent upon IKK α/β [57]. Thus, RIPK1 can be inhibited in the cytosol by MAPK-activated protein kinase 2 (MK2) independent of the activity of IKK α/β [58,59]. DHA has been shown to specifically inhibit IKK α/β and p38 [55,56]. In view of the role of IKK and the p38 MAPK-MK2 pathway in regulating survival function [[57], [58], [59]], it is conceivable that DHA may sensitize the kinase-dependent cell death function of RIPK1, inducing inactivation of MK2 by inhibiting p38. Our results also show that AA preconditions cells for necroptosis because AA is an inhibitor of apoptosis. Thus, under pathophysiological conditions in which AA is oxidized to form DHA, DHA causes necroptotic cell death to be favored by preventing apoptosis and sensitizing RIPK1-kinase activity. This dual effect of vitamin C on survival would be dramatic in cells that have a low apoptotic rate and that face acute oxidative stress conditions if they are able to accumulate a high concentration of vitamin C. Unfortunately, neurons accumulate more AA under physiological conditions (up to 10 mM [1]) and are refractory to apoptosis [33,34,60]. In conclusion, under pathophysiological conditions where the oxidation of neuronal AA is favored, such as stroke, trauma, Alzheimer's disease, Huntington's disease, or Parkinson's disease, DHA could induce cell death via necroptosis. Interestingly, our data suggest that SVCT2 exerts physiological functions, ultimately controlling cerebral oxidative stress given that its overexpression decreased the rate of cell death compared with wild-type cells. In contrast, SVCT2^−/−^ neurons were highly sensitive to oxidative stress, suggesting that SVCT2 could be a marker of resistance to cell death in the brain.

## Author contributions

LF, MJB, PV, KS and FN conceived the experiments. LF performed the experiments; LF, MJB, PV, KS and FN analyzed the data; LF, PV and FN wrote the article; FN, KS and PV critically revised the manuscript. All authors approved the final version of the manuscript.

## Declarations of competing interest

None.

## Appendix A Supplementary data

Data Profile Data Profile
