# Supplementary material for: Vitamin C controls neuronal necroptosis under oxidative stress
Source: Redox Biol. 2019 Dec 16;29:101408. doi: 10.1016/j.redox.2019.101408 (PMC6938857; doi:10.1016/j.redox.2019.101408)
Supplement: Supplementary Information [file mmc2.docx]

**Supplementary Information.**

**VITAMIN C CONTROLS NEURONAL NECROPTOSIS UNDER OXIDATIVE STRESS**

Luciano Ferrada, Maria Jose Barahona, Katterine Salazar, Peter Vandenabeele, and Francisco Nualart*.

*Address correspondence and reprint requests to F. Nualart, Departamento de Biología Celular, Facultad de Ciencias Biológicas, Universidad de Concepción, Casilla 160-C, Concepción, Chile

E-mail: [frnualart@udec.cl](mailto:fnualart@udec.cl)

**Table 1.**

| **REAGENT or RESOURCE** | SOURCE | IDENTIFIER |
| --- | --- | --- |
| **Antibodies** | | |
| Anti-RIPK1 | Cell Signaling Technology | Cat#D94C12 |
| anti-phospho-MLKL | Cell Signaling Technology | Cat#D6H3V |
| Anti-GLUT1 | Merck Millipore | Cat#07-1401 |
| anti-RIPK3 | Merck Millipore | Cat#MABC28 |
| anti-MLKL | Merck Millipore | Cat#MABC604 |
| Anti- β-Actin | Santa Cruz Biotechnology | Cat#sc-47778 |
| anti-SVCT2 | Novus Biologicals | Cat#NBP2-13319 |
| **Chemicals** |  |  |
| Nec-1 | Abcam | Cat#ab141053 |
| zVAD.FMK | Abcam | Cat#ab120382 |
| Mdivi | Abcam | Cat#ab144589 |
| NAC | Abcam | Cat#ab143032 |
| Nec-1s | Biovision | Cat#2263 |
| Hoechst 33342 | Life Technologies | Cat#H1399 |
| MitotrackerCMXRos | Life Technologies | Cat#M7512 |
| Cellmask | Life Technologies | Cat#C10046 |
| CellROX Deep Red | Life Technologies | Cat#C10422 |
| Phalloidin | Life Technologies | Cat#A12379 |
| TOPRO-3 | Life Technologies | Cat#T3605 |
| hTNFa | Life Technologies | Cat#10602HNAE5 |
| Lipofectamine 3000 | Life Technologies | Cat#L3000015 |
| L-AA | Sigma | Cat#A4544 |
| **Bacterial and Virus Strains** | | |
| Lentivirus-hSVCT2wt-EYFP | Salazar et al., 2016 | N/A |
| Lentivirus-EGFP | Salazar et al., 2016 | N/A |
| **Critical Commercial Assays** | | |
| XTT | Biological industries | Cat#20-300-1000 |
| FRASC | Bioassay system | Cat#EASC-100 |
| Trizol | Invitrogen | Cat#15596018 |
| Brilliant II SYBR® Green | Agilent | Cat#600828 |
| **Experimental Models: Cell Lines** | | |
| N2a-hSVCT2wt-EYFP | This study | N/A |
| N2a-EGFP | This study | N/A |
| HN33.11-hSVCT2wt-EYFP | This study | N/A |
| HN33.11-EGFP | This study | N/A |
| N2a*^(Mlkl-/-)^* | This study | N/A |
| N2a*^(Svct2-/-)^* | This study | N/A |
| Rat Cortical Neurons | This study | N/A |
| **Oligonucleotides** |  |  |
| Primers for qPCR see table 2 | This study | N/A |
| **Plasmid and sgRNA sequence** | | |
| CAG-Cas9-2a-RFP, Cas9-ElecD | Atum | Cat#pD1321-AP |
| SVCT2-TGTAGATCATATCCGACCTC | N/A | N/A |
| MLKL-GCACACGGTTTCCTAGACGC | N/A | N/A |
| Software and Algorithms | | |
| Imaris v 9.1 | Bitplane | <https://imaris.oxinst.com/> |
| Flowjo | FlowJo LLC | <https://www.flowjo.com/> |
| Image J | NIH | <https://imagej.nih.gov/ij/?> |
| GraphPad Prism version 6.01. | Graphpad | <https://www.graphpad.com/scientific-software/prism/> |
| Zen | Zeiss | <https://www.zeiss.com/microscopy/int/products/microscope-software/zen-lite.html> |

**Table 2.**

| **Target** | **Forward 5´🡪3´** | **Reverse 5´🡪3´** |
| --- | --- | --- |
| mBAX | AGGATGCGTCCACCAAGAAG | TTGGATCCAGACAAGCAGCC |
| mBcl-2 | CTGGATCCAGGATAACGGAGG | GCAGCAAGCTACTCAGACGA |
| mCasp8 | CTGATAAGGTGCTATCTTGGGT | GTCCAAGCACAGGAACTTGAG |
| mRIPK1 | TCTCTGGGCCAGTAGCAGAT | CGGGCACAGTTTTTCCACTG |
| mRIPK3 | CCAGAGAGCCAAGCCAAAGAG | AGCCACGGGGTCAGAAGATGT |
| mMLKL | GGAACTGAGCGCAGGATAGA | ACACGGTTTCCTAGACGCTG |
| rBAX | GCGAATTGGCGATGAACTGG | GTGTCCAGCCCATGATGGTT |
| rBcl-2 | GAGGGGCTACGAGTGGGATA | CGGTAGCGACGAGAGAAGTC |
| rCasp8 | CAAACCTCGGGGGTACTGTC | ATCCGTTCCGTAGACGATGC |
| rRIPK1 | AATGGGACCTGTGGAGGAGT | GGAGACCCTTCGCTTCCTTT |
| rRIPK3 | CCAGAGAGCCAAGCCAAAGAG | AGCCACGGGGTCAGAAGATGT |
| mr SVCT2 | TGCCAGGAAGGGTGTACTTC | CCGGTACCAAATATGCCATC |
| rGLUT1 | TCAAACATGGAACCACCGC | AGAAACCCATAAGCACGGCA |
| Cyclophilin | ATAATGGCACTGGTGGCAAGTC | ATTCCTGGACCCAAAACGCTCC |


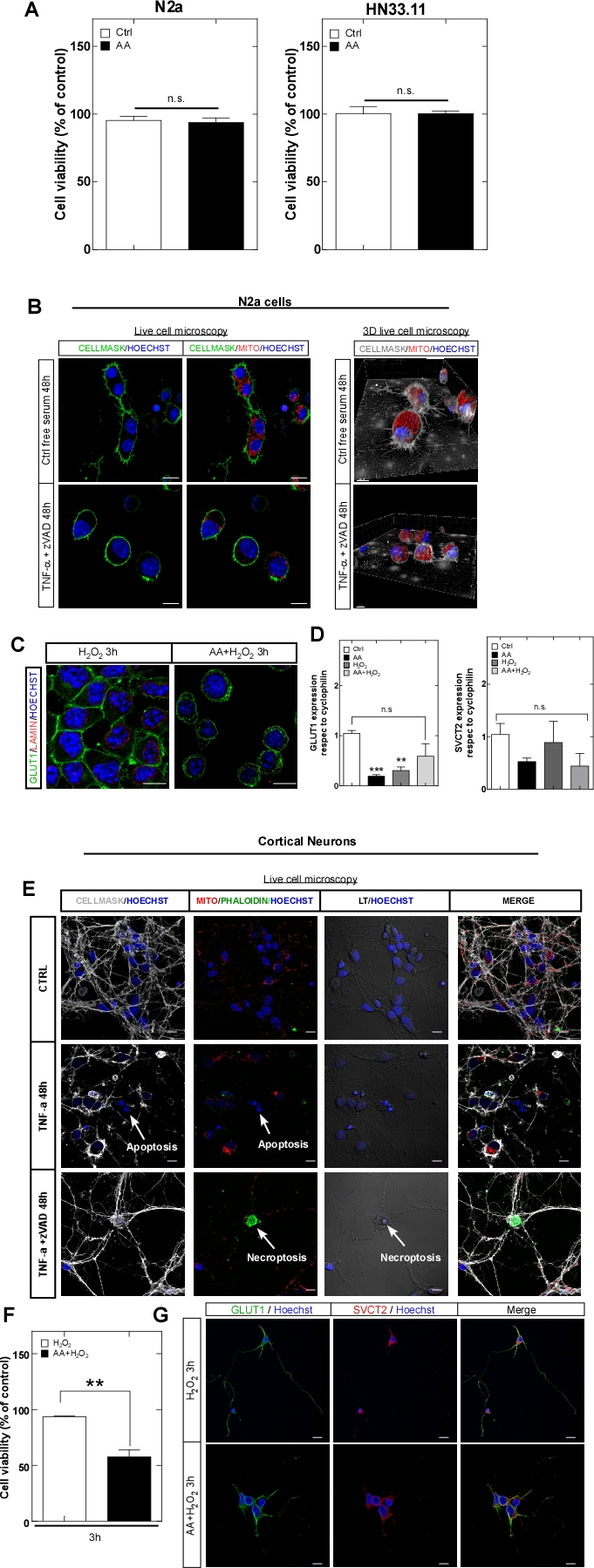


**Supplementary Fig. 1. Features of neuronal disintegration during cell death. Related to Fig. 1.** (A) Cell viability analysis of N2a and HN33.11 supplemented with 200 µM AA every 12 h for 36 h. (B, E) Morphological characterization during pharmacological neuronal necroptosis. (C) GLUT1 distribution to the perinuclear zone. (D) mRNA levels of GLUT1 and SVCT2. n=4 biologically independent samples. Cells were assayed in triplicate at each condition. Scale bar is 10 µm for N2a cells and 5 µm for cortical neurons. (F) Cell viability analysis of cortical neurons supplemented with 200 µM AA every 12 h for 36 h and oxidative stress induction with 30 µM H_2_O_2._ (G) Codistribution of GLUT1 and SVCT2 in cortical neurons. Data were analyzed by Student’s *t*-test (two-tailed) and are presented as the mean ± SEM; all data are representative of 3 separate experiments. * *p*≤0.05, ** *p*≤0.01, *** *p*≤0.001.


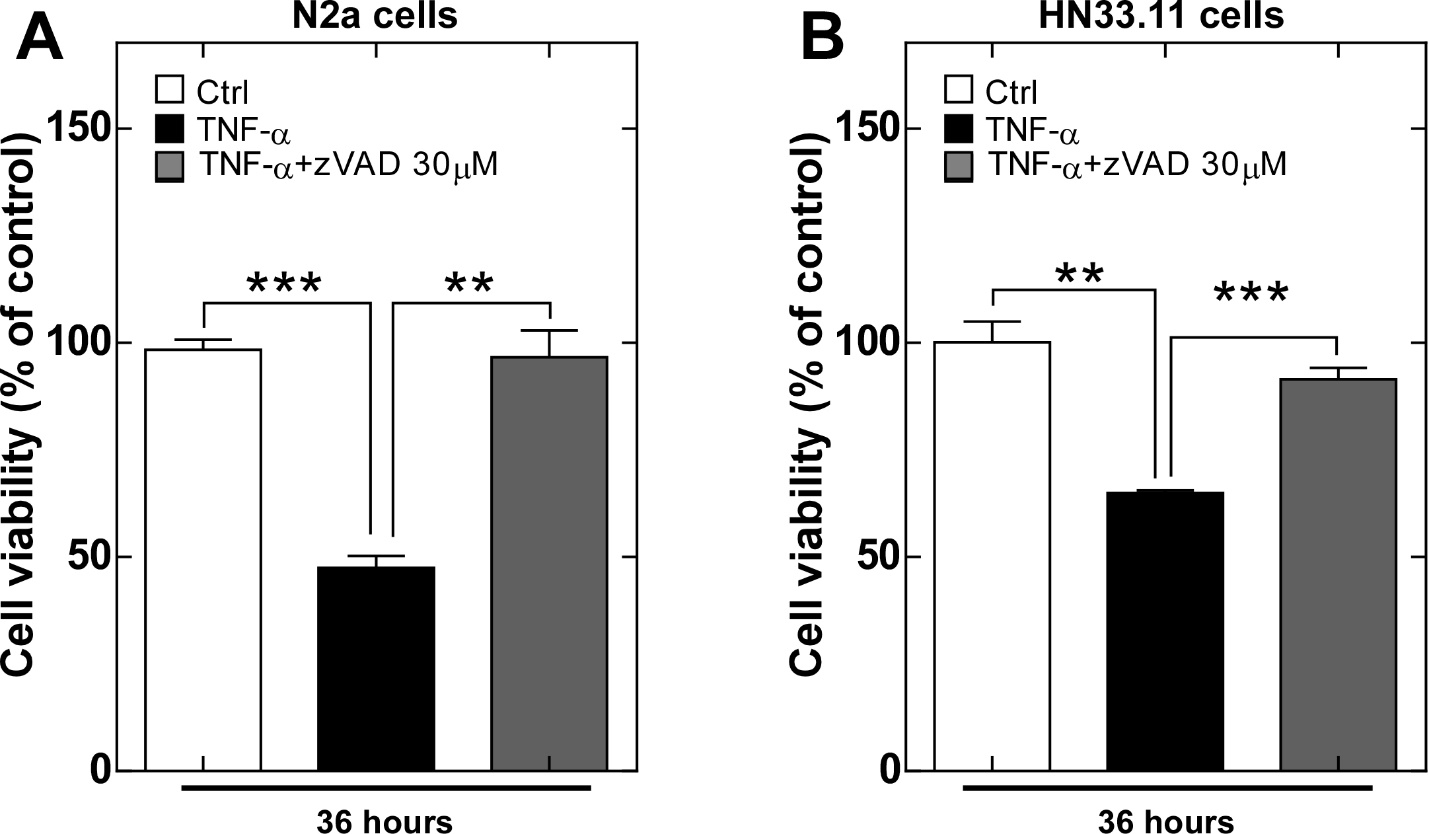


**Supplementary Fig. 2. Inhibition of neuronal apoptosis induced by TNF-alpha using zVAD. Related to Fig. 2.** (A, B) Cell viability analysis of N2a and HN33.11 cells by treatment with TNF-a (10 ng/mL) and 30 µM zVAD. n=3 biologically independent samples. Cells were assayed in triplicate at each condition. Data were analyzed by Student’s *t*-test (two-tailed) and are presented as the mean ± SEM; all data are representative of three separate experiments. * *p*≤0.05, ** *p*≤0.01, *** *p*≤0.001.


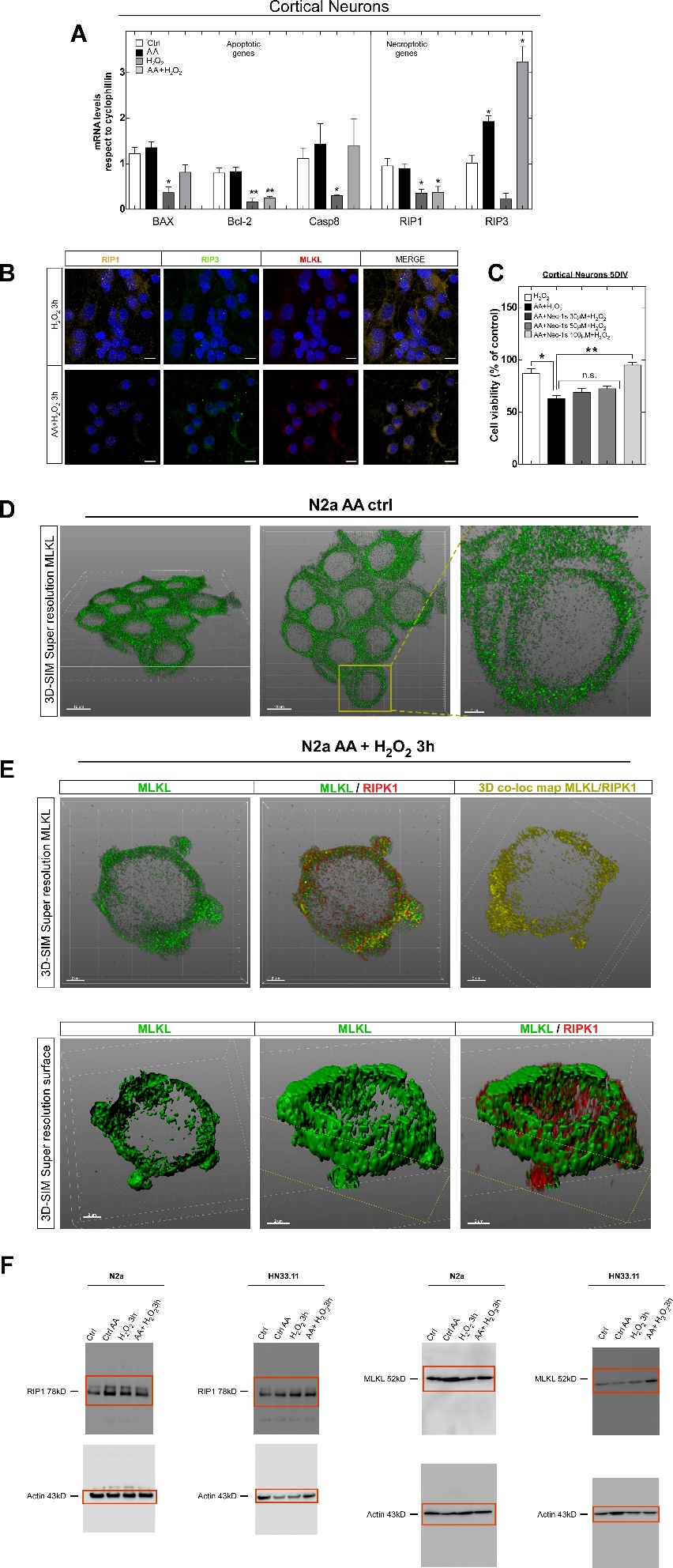


**Supplementary Fig. 3. Vitamin C regulates the expression of RIPK1, RIPK3 and MLKL, stimulating necroptosis in cortical neurons, while AA oxidation induces bubble formation in the plasma membrane in N2a cells. Related to Fig. 3.**

(A) mRNA levels of apoptotic and necroptotic genes in cortical neurons, n=3 biologically independent samples. (B) Analysis of the expression and localization of RIPK1, RIPK3 and MLKL in cortical neurons. (C, D) Necroptosis inhibition with necrostatin-1s (n=3 biologically independent samples). (E) Redistribution of MLKL. (F) 3D-SIM microscopy analysis of RIPK1 and MLKL colocalization. The formation of bubbles in N2a cells in response to the oxidation of AA is also shown. (G) Original gels of Fig. 3. Scale bar, SR 2 µm, cortical neurons 5 µm. Cells were assayed in triplicate at each condition. Data are shown as the mean ± SEM (two-tailed Student's t-tests); all data are representative of three separate experiments. * *p*≤0.05, ** *p*≤0.01, *** *p*≤0.001.


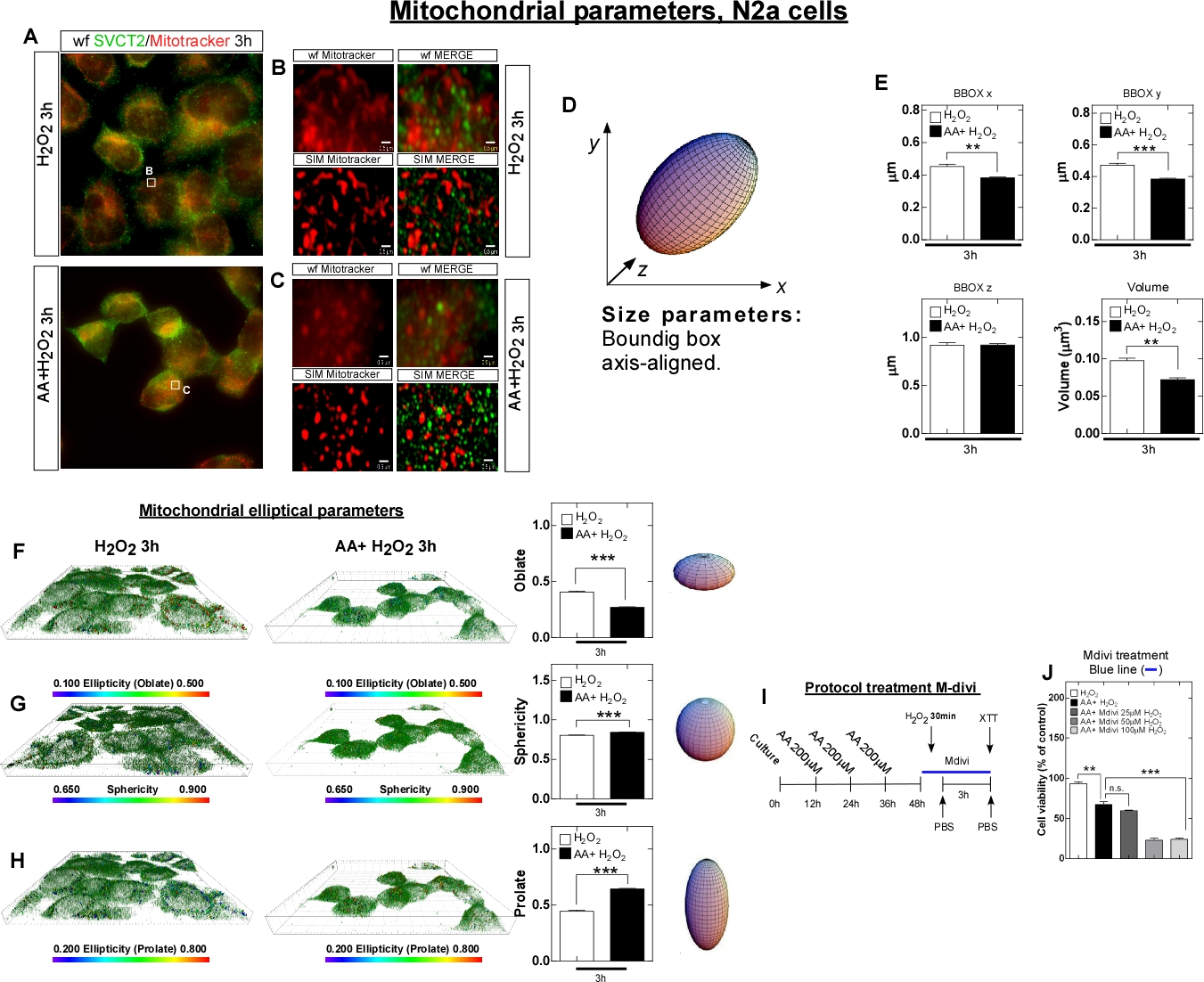


**Supplementary Fig. 4. The oxidation of vitamin C alters the size and neuronal mitochondrial morphology; however, the inhibition of Drp-1 does not prevent cell death. Related to Fig. 5.** A. Wide field microscopy. (B, C) SIM superresolution microscopy. (D) Bounding box axis-aligned scheme. (E) Mitochondrial size parameters. n=4 biologically independent samples. (F, G, H) Mitochondrial elliptic parameters. n=4 biologically independent samples. (I) Scheme of Mdivi treatment. (J) Cell viability analysis at 3 h posttreatment. n=3 biologically independent samples. MitotrackerCMXRos at 50 nM was used as a mitochondrial marker. Scale bar, 500 nm. Cells were assayed in triplicate at each condition. Data are shown as the mean ± SEM (two-tailed Student's t-tests); all data are representative of three separate experiments. * *p*≤0.05, ** *p*≤0.01, *** *p*≤0.001.


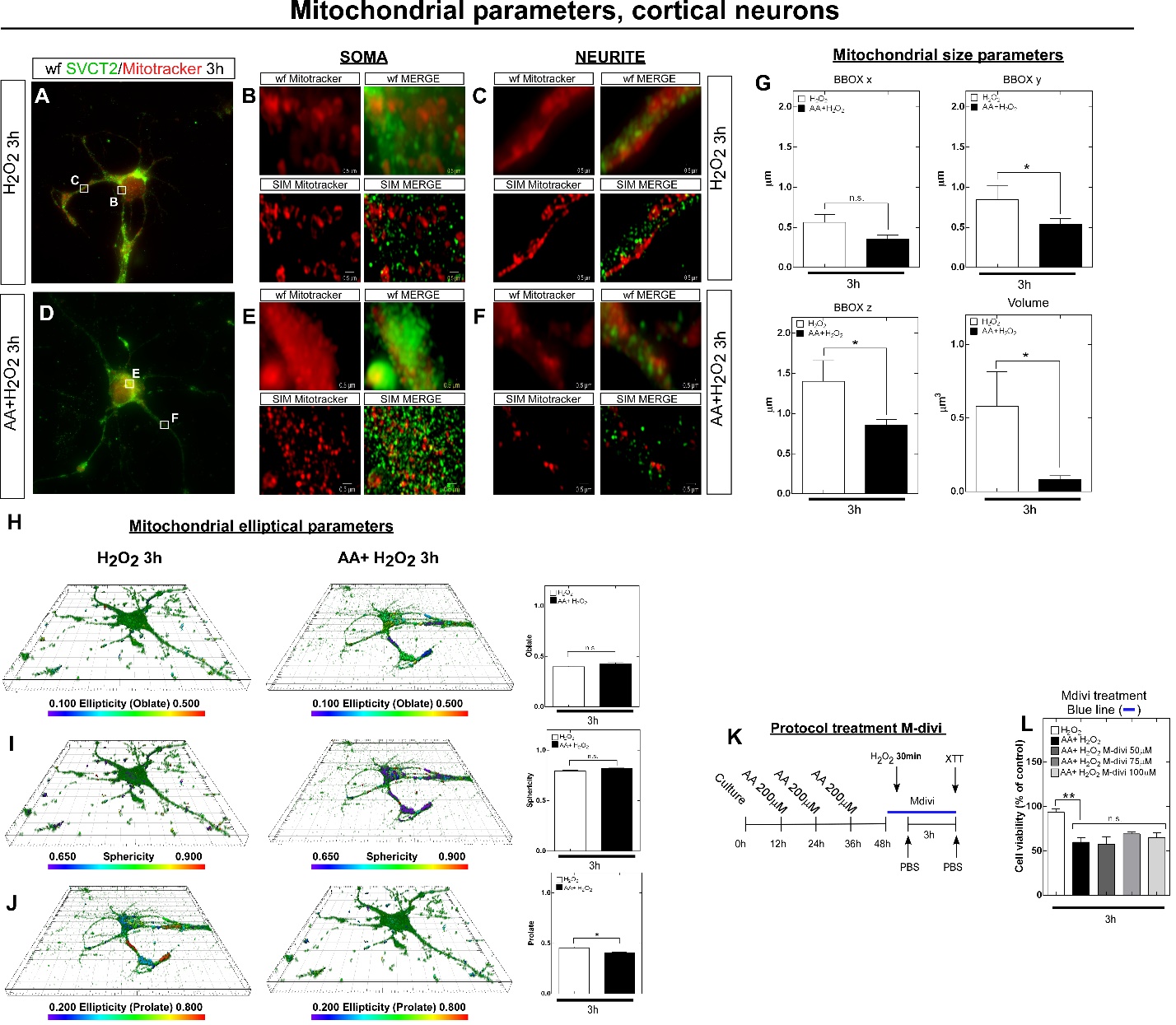


**Supplementary Fig. 5. In cortical neurons, the oxidation of vitamin C alters the size and mitochondrial morphology; however, the inhibition of Drp-1 does not prevent cell death. Related to Fig. 5.** (A, B) Wide-field microscopy. (B, C, E, F) SIM superresolution microscopy. (G) Mitochondrial size parameters (n=4). (H, I, J) Mitochondrial elliptic parameters (n=4). (K) Scheme of Mdivi treatment. (L) Quantification of cell viability in cortical neurons (n=3 biologically independent samples). MitotrackerCMXRos at 50 nM was used as a mitochondrial marker. Scale bar, 500 nm. Cells were assayed in triplicate at each condition. Data are shown as the mean ± SEM (two-tailed Student's t-tests); all data are representative of three separate experiments. * *p*≤0.05, ** *p*≤0.01, *** *p*≤0.001.

**
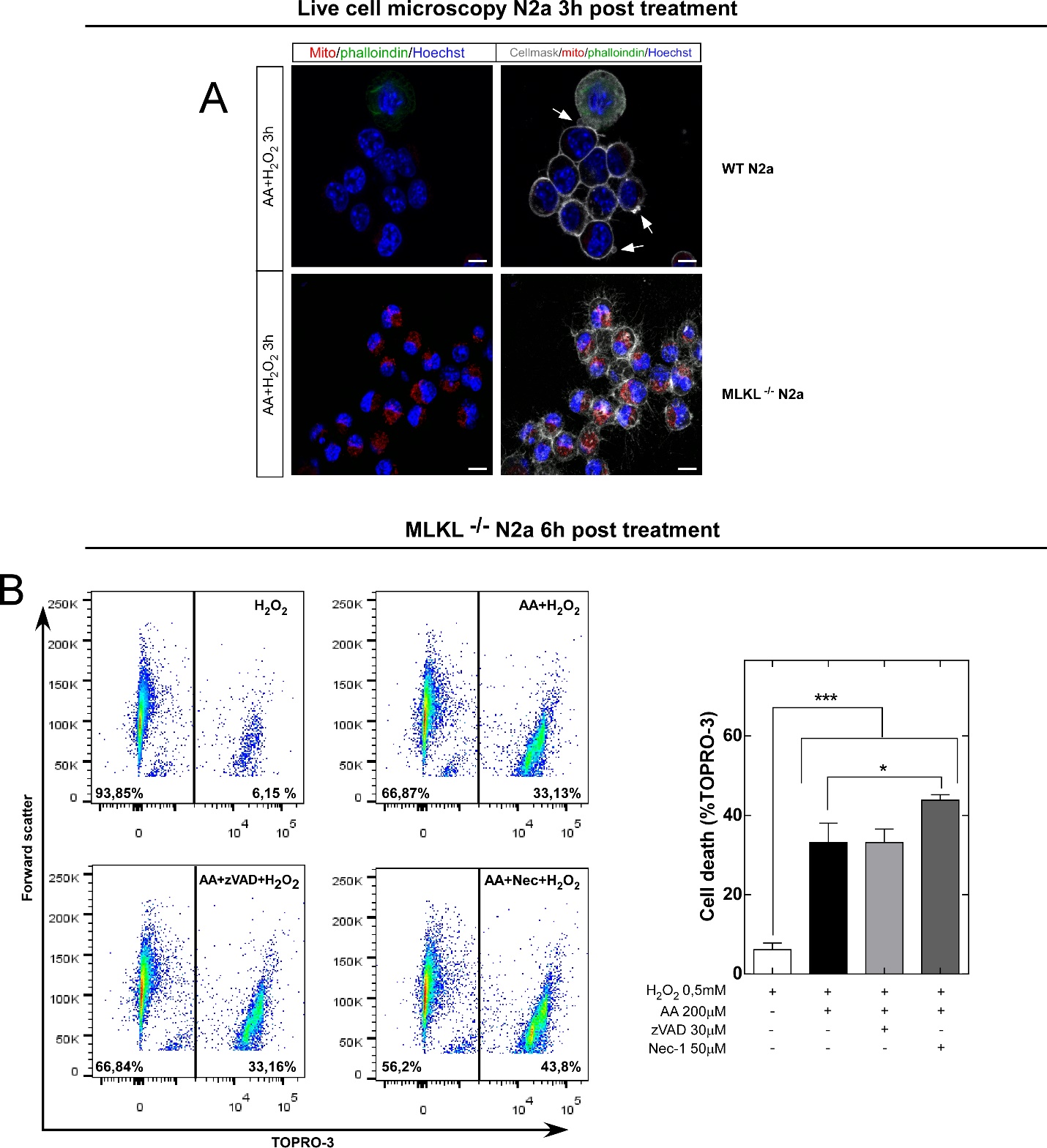
**

**Supplementary** **Fig. 6. Loss of MLKL only delays neuronal death induced by AA oxidation. Related to Fig. 7.** (A) Live-cell microscopy analysis. Arrows indicate the formation of bubbles in N2a wild-type cells. (B) Quantification of cell death. n=3 biologically independent samples. Cells were assayed in triplicate at each condition. Scale bar, 10 µm. Data are shown as the mean ± SEM (two-tailed Student's t-tests); all data are representative of three separate experiments. * *p*≤0.05, ** *p*≤0.01, *** *p*≤0.001.

**Description videos**

**Video 1.** 3D animation (Imaris software) of N2a cells treated with AA at 3 h posttreatment with H_2_O_2_. GLUT1 (green) was used as a cellular marker.

**Video 2.** 3D reconstruction (Imaris software) of N2a cells at 3 h after treatment with H_2_O_2_. GLUT1 (green) was used as a cellular marker.

**Video 3.** 3D animation (Imaris software) of N2a cells treated with TNF-α + zVAD for 48 h. Cellmask (green) was used as a marker for the plasma membrane.

**Video 4.** 3D animation (Imaris software) of cortical neurons treated with TNF-α + zVAD for 48 h. Cellmask (green) was used as a marker for the plasma membrane.

**Video 5.** Disintegration characteristics during neuronal necroptosis in N2a cells determined by real-time live-cell microscopy. The probes used were Cellmask as a plasma membrane marker (white), Mitotracker CMXRos as a mitochondrial marker (red), Hoechst as a nuclear stain and phalloidin (green) as a marker of membrane integrity.

**Video 6.** Disintegration characteristics during neuronal necroptosis in HN33.11 cells determined by real-time live-cell microscopy. The probes used were Cellmask as a plasma membrane marker (white), Mitotracker CMXRos as a mitochondrial marker (red), Hoechst as a nuclear stain and phalloidin (green) as a marker of membrane integrity.

**Video 7.** Disintegration characteristics during neuronal necroptosis in cortical neurons determined by real-time live-cell microscopy. The probes used were Cellmask as a plasma membrane marker (white), Mitotracker CMXRos as a mitochondrial marker (red), Hoechst as a nuclear stain and phalloidin (green) as a marker of membrane integrity.

**Video 8.** Disintegration characteristics during neuronal necroptosis in N2a cells over a long period of time determined by real-time live-cell microscopy. The probes used were Mitotracker CMXRos as a mitochondrial marker (red), TOPRO-3 (blue) and phalloidin (green) as a marker of the integrity of the plasma membrane.
